# Predictive risk factors for distant metastasis in pediatric differentiated thyroid cancer from Saudi Arabia

**DOI:** 10.3389/fendo.2023.1228049

**Published:** 2023-10-06

**Authors:** Sandeep Kumar Parvathareddy, Abdul K. Siraj, Padmanaban Annaiyappanaidu, Nabil Siraj, Maha Al-Rasheed, Wael Al-Haqawi, Zeeshan Qadri, Khawar Siddiqui, Saif S. Al-Sobhi, Fouad Al-Dayel, Khawla S. Al-Kuraya

**Affiliations:** ^1^ Human Cancer Genomic Research, Research Center, King Faisal Specialist Hospital and Research Centre, Riyadh, Saudi Arabia; ^2^ Department of Pediatric Hematology-oncology, King Faisal Specialist Hospital and Research Centre, Riyadh, Saudi Arabia; ^3^ Department of Surgery, King Faisal Specialist Hospital and Research Centre, Riyadh, Saudi Arabia; ^4^ Department of Pathology, King Faisal Specialist Hospital and Research Centre, Riyadh, Saudi Arabia

**Keywords:** differentiated thyroid cancer, pediatric, distant metastasis, lung metastasis, risk factors

## Abstract

**Background:**

Despite their excellent prognosis, children and young adults (CAYA) with differentiated thyroid cancer (DTC) tend to have more frequent occurrence of distant metastasis (DM) compared to adult DTC. Data about DM in CAYA from Middle Eastern ethnicity is limited.

**Methods:**

Medical records of 170 patients with DTC ≤18 years were retrospectively reviewed. Clinico-pathological factors associated with lung metastasis in CAYA, their clinical presentation and outcome were analyzed. Rick factors related to distant metastasis-free survival (DMFS) for the whole cohort were evaluated.

**Results:**

DM was observed in 27 patients and all were lung metastasis. Lung metastasis was significantly associated with younger age (≤15 years), extrathyroidal extension (ETE), multifocal tumors, bilaterality, presence of lymph node (LN) disease and high post-operative stimulated thyroglobulin (sTg). Highest negative predictive values were seen with low post-operative sTg (97.9%), absence of LN disease (93.8%), absence of ETE (92.2%) and age older than 15 years (92.9%). Post-therapy whole body scan (WBS) identified most of the lung metastasis (21 of 27; 77.8%). Upon evaluating patients response according to ATA guidelines, excellent response was seen in only one patient, while biochemical persistence and structural persistence were seen in 11.1% (3/27) and 77.8% (21/27), respectively. Elevated post-operative sTg (>10ng/ml) was the only risk factor found to be significantly associated with both biochemical persistence (with or without structural persistence (p = 0.0143)) and structural persistence (p = 0.0433). Cox regression analysis identified age and post-operative sTg as independent risk factors related to DMFS. Based on these two risk factors for DMFS, patients were divided into 3 groups: low risk (no risk factors), intermediate risk (1 risk factor) and high risk (both risk factors). 20-year DMFS rates in the low-, intermediate- and high-risk groups were 100.0%, 81.3% and 23.7% respectively (p < 0.0001).

**Conclusion:**

Higher suspicion for metastatic pediatric DTC should be considered in patients who are young, have LN disease, extrathyroidal extension and elevated post-operative sTg. Persistent disease, despite therapy, is very common and it appears to be related to post-operative sTg level. Hence, risk adaptive management is desirable in CAYA with DTC.

## Introduction

Differentiated thyroid cancer (DTC) is a common solid tumor in children and young adults (CAYA) (1, 2). The incidence of CAYA DTC has been increasing lately (3). Despite similarity in pathophysiology, pediatric and adult DTC have different clinical behavior. DTC in CAYA tend to present with more aggressive clinical features at diagnosis such as large tumor size and high proportion of bilateral and multifocal tumors (4, 5). Furthermore, children and adolescents with DTC are more likely to present with advanced disease with higher rate of recurrence, persistence, regional and distant metastasis (6, 7).

Even so, the reported mortality rate in very low compared to adult, with a disease specific survival of 99% in affected children (6, 8). The optimal approach to evaluation and treatment of CAYA with distant metastasis and particularly lung metastasis, remains unresolved (9). According to the American Thyroid Association (ATA) treatment guidelines (10), total thyroidectomy is recommended for all pediatric DTC patients. In clinical practice, pediatric DTC patients are generally treated with surgery followed by possible radioactive iodine (RAI) therapy, which is usually given in the context of post-operative staging based on diagnostic thyroid scan and stimulated thyroglobulin (sTg) level (10, 11), especially in cases of known distant metastatic pediatric DTC. The lungs are the primary site of DM, detected in up to 20% of childhood DTC (6, 7, 12). Despite the fact that most pediatric patients with DM do not reach remission, they do have an extremely low disease specific mortality (7, 9, 12–14).

The management of pediatric DTC with DM is challenging and recent reports have highlighted the importance of personalized therapy, based on the risk of poor outcome, to maintain the balance between treating aggressive disease and minimizing the potential long-term sequelae of over-zealous therapy in children with DTC (15–17). Therefore, it is extremely important to identify the subset of pediatric DTC who are at risk of poor clinical outcome, especially those with distant metastasis, where data regarding CAYA DTC from Middle Eastern ethnicity is limited.

In the present study, we retrospectively analyzed a large cohort of children and young adults with distant metastatic DTC, treated at a single tertiary care hospital in Saudi Arabia. Prevalence, predictive markers and response to therapy of CAYA with distant metastatic DTC were evaluated. Risk factors for DM were evaluated and risk stratification was attempted.

## Materials and methods

### Patient selection

One hundred and seventy CAYA (≤ 18 years) DTC patients diagnosed between 1988 and 2018 at King Faisal Specialist Hospital and Research Centre (Riyadh, Saudi Arabia) were included in the study. Cases were identified based on clinical history followed by fine needle aspiration biopsy for confirmation. The Institutional Review Board of the hospital approved this study and since only retrospective patient data were used, the Research Advisory Council (RAC) provided waiver of consent under project RAC # 221 1168 and # 2110 031. The study was conducted in accordance with the Declaration of Helsinki.

### Clinico-pathological and follow-up data

Baseline clinico-pathological data were collected from case records and have been summarized in **Table 1**. Staging of DTC was performed using the eighth edition of American Joint Committee on Cancer (AJCC) staging system (18). The patients were seen 6 to 8 weeks after surgery, at which time, a diagnostic radioactive iodine (I-123) whole body scan (DxWBS) and neck ultrasonography were performed, and stimulated thyroglobulin (sTg), anti-Tg antibodies, TSH and free T4 were measured. Radioactive iodine (I-131) was administered at activities that averaged 150 to 200 mCi for a 70-kg patient, adjusted for body weight, with consideration of the extent of lung involvement found on the DxWBS. The initial I-131 therapies were administered every 6 to 12 months. Monitoring of these patients was achieved by periodic (every 3 to 9 months) measurement of TSH, FT4, Tg, and anti-Tg antibodies, neck ultrasonography, DxWBS, and CT scan of the lungs.

**Table 1:** Clinico-pathological characteristics and associations of CAYA DTC with and without lung metastasis.

	Total (n = 170)	M0 (n = 143)	M1 (n = 27)	p value	Sens	Spec	PPV	NPV
**Age at diagnosis, years (mean ± SD)**	14.9 ± 3.0	15.1 ± 3.0	13.7 ± 2.7	0.0253				
≤ 15 years	86 (50.6%)	65 (45.5%)	21 (77.8%)	0.0016	77.8%	54.6%	24.4%	92.9%
16 – 18 years	84 (49.4%)	78 (54.5%)	6 (22.2%)					
Gender								
Male	43 (25.3%)	35 (24.5%)	8 (29.6%)	0.5774	29.6%	75.5%	18.6%	85.0%
Female	127 (74.7%)	108 (75.5%)	19 (70.4%)					
**Tumor diameter (cm)**	3.3 ± 1.8	3.1 ± 1.6	3.9 ± 2.3	0.0572				
Histologic subtype								
FTC	13 (7.7%)	13 (9.1%)	0	0.0302				
PTC	157 (92.3%)	130 (90.9%)	27 (100%)					
PTC subtypes								
Classical variant	103 (65.6%)	88 (67.7%)	15 (55.6%)	0.3001				
Follicular variant	26 (16.6%)	20 (15.4%)	6 (22.2%)					
Tall cell variant	8 (5.1%)	7 (5.4%)	1 (3.7%)					
Diffuse sclerosing variant	10 (6.4%)	6 (4.6%)	4 (14.8%)					
Solid variant	4 (2.5%)	3 (2.3%)	1 (3.7%)					
Oncocytic variant	2 (1.3%)	2 (1.5%)	0					
Columnar cell variant	1 (0.6%)	1 (0.8%)	0					
Mixed	3 (1.9%)	3 (2.3%)	0					
Tumor laterality								
Unilateral	111 (67.7%)	98 (71.5%)	13 (48.2%)	0.0209	51.8%	71.5%	26.4%	88.3%
Bilateral	53 (32.3%)	39 (28.5%)	14 (51.8%)					
Tumor focality								
Unifocal	79 (47.9%)	71 (51.5%)	8 (29.6%)	0.0352	70.4%	51.5%	22.1%	89.9%
Multifocal	86 (52.1%)	67 (48.5%)	19 (70.4%)					
Extrathyroidal extension								
Present	79 (50.6%)	58 (45.0%)	21 (77.8%)	0.0015	77.8%	55.0%	26.6%	92.2%
Absent	77 (49.4%)	71 (55.0%)	6 (22.2%)					
pT								
T1 and T2	99 (58.2%)	87 (60.8%)	12 (44.4%)	0.1823	45.5%	69.1%	20.4%	87.9%
T3 and T4	49 (28.8%)	39 (27.3%)	10 (37.1%)					
pN								
N0	32 (22.9%)	30 (26.3%)	2 (7.7)	0.0413	92.3%	26.3%	22.2%	93.8%
N1	108 (77.1%)	84 (73.7%)	24 (92.3%)					
Pre-operative Tg								
High	15 (31.9%)	9 (22%)	6 (100%)	0.0005	100%	78.1%	40%	100%
Low	32 (68.1%)	32 (78%)	0					
Post-operative sTg								
High	96 (66.7%)	75 (61.5%)	21 (95.4%)	0.0004	95.4%	38.5%	21.9%	97.9%
Low	48 (33.3%)	47 (38.5%)	1 (4.6%)					
*BRAF* mutation								
Present	28 (23.9%)	28 (28.6%)	0	0.0062	0%	71.4%	0%	78.6%
Absent	89 (76.1%)	70 (71.4%)	19 (100%)					
*NRAS* mutation								
Present	4 (4.0%)	4 (4.7%)	0	1.0000	0%	95.3%	0%	84.4%
Absent	96 (96.0%)	81 (95.3%)	15 (100.0%)					
*HRAS* mutation								
Present	0	0	0	–	–	–	–	–
Absent	103 (100.0%)	87 (100.0%)	16 (100.0%)					
*KRAS* mutation								
Present	0	0	0	–	–	–	–	–
Absent	101 (100.0%)	85 (100.0%)	16 (100.0%)					
*TERT* mutation								
Present	1 (0.9%)	1 (0.9%)	0	1.0000	0%	98.9%	0%	83.8%
Absent	105 (99.1%)	88 (98.9%)	17 (100%)					
*NTRK3* fusion								
Present	8 (11.8%)	4 (7.3%)	4 (30.8%)	0.0379	30.8%	92.7%	50%	85%
Absent	60 (88.2%)	51 (92.7%)	9 (69.2%)					

CAYA, Children and young adults; DTC, Differentiated thyroid cancer; SD, Standard deviation; cm, centimeter; FTC, Follicular thyroid cancer; PTC, Papillary thyroid cancer; sTg, stimulated thyroglobulin.

Response to therapy was defined as ‘‘excellent,’’ with basal (unstimulated) Tg <0.2 ng/mL or thyrotropin-stimulated Tg (sTg) <1 ng/mL (with normal anti-Tg titers) and no structural disease on imaging; biochemical persistence, with abnormal biochemistry and no structural disease; structural persistence, with disease seen on imaging (regardless of Tg and anti-Tg levels); or indeterminate, wherein anti-Tg was elevated or data were missing (19).

### DNA isolation

DNAs were extracted from PTC formalin-fixed and paraffin-embedded (FFPE) tumor tissues utilizing Gentra DNA isolation kit (Gentra, Minneapolis, MN, USA) according to manufacturer’s protocols as elaborated in the previous studies (20).

### Sanger sequencing analysis

PCR and Sanger sequencing analysis of hotspot mutations in *BRAF*, *KRAS*, *HRAS*, *NRAS* and promoter region in *TERT* gene were carried out as described previously (21). Primer 3 online software was utilized to design the primers (available upon request). Reference sequences were downloaded from NCBI GenBank. Sequencing results were compared with the reference sequence by Mutation Surveyor V4.04 (Soft Genetics, LLC, State College, PA).

### Fluorescence *in situ* hybridization analysis

FISH assay for NTRK3 gene fusions was performed as described previously on a tissue microarray format following the manufacturer’s instructions (22). NTRK3 dual-color break-apart probe (Empire Genomics, Williamsville, NY, USA) were utilized. NTRK3 fusion was termed as positive if >20% of tumor cells showed split red and green signals or a single signal in addition to a single fused signal; otherwise, the specimen was classified as fusion negative (22).

### Statistical analysis

The associations between clinico-pathological variables and distant metastases was performed using contingency table analysis and Chi square/Fisher exact tests. Unpaired t tests were used for comparisons of continuous variables. Distant metastasis-free survival (DMFS) was the primary endpoint and progression-free survival (PFS) was the secondary endpoint. DMFS and PFS rates were calculated by the Kaplan-Meier method. Cox proportional hazards model was used for analyzing the impact of prognostic factors on DMFS and PFS in univariate and multivariate manner. Risk stratification was performed according to the factors related to survival. Two-sided tests were used for statistical analyses with a limit of significance defined as p value < 0.05. Data analyses were performed using the JMP14.0 (SAS Institute, Inc., Cary, NC) software package.

Receiver operating characterisitcs (ROC) curve analysis was performed using MedCalc software, version 10.4.7.0 for Windows (MedCalc, Ostend, Belgium).

## Results

### Patient and tumor characteristics

**Figure 1:**
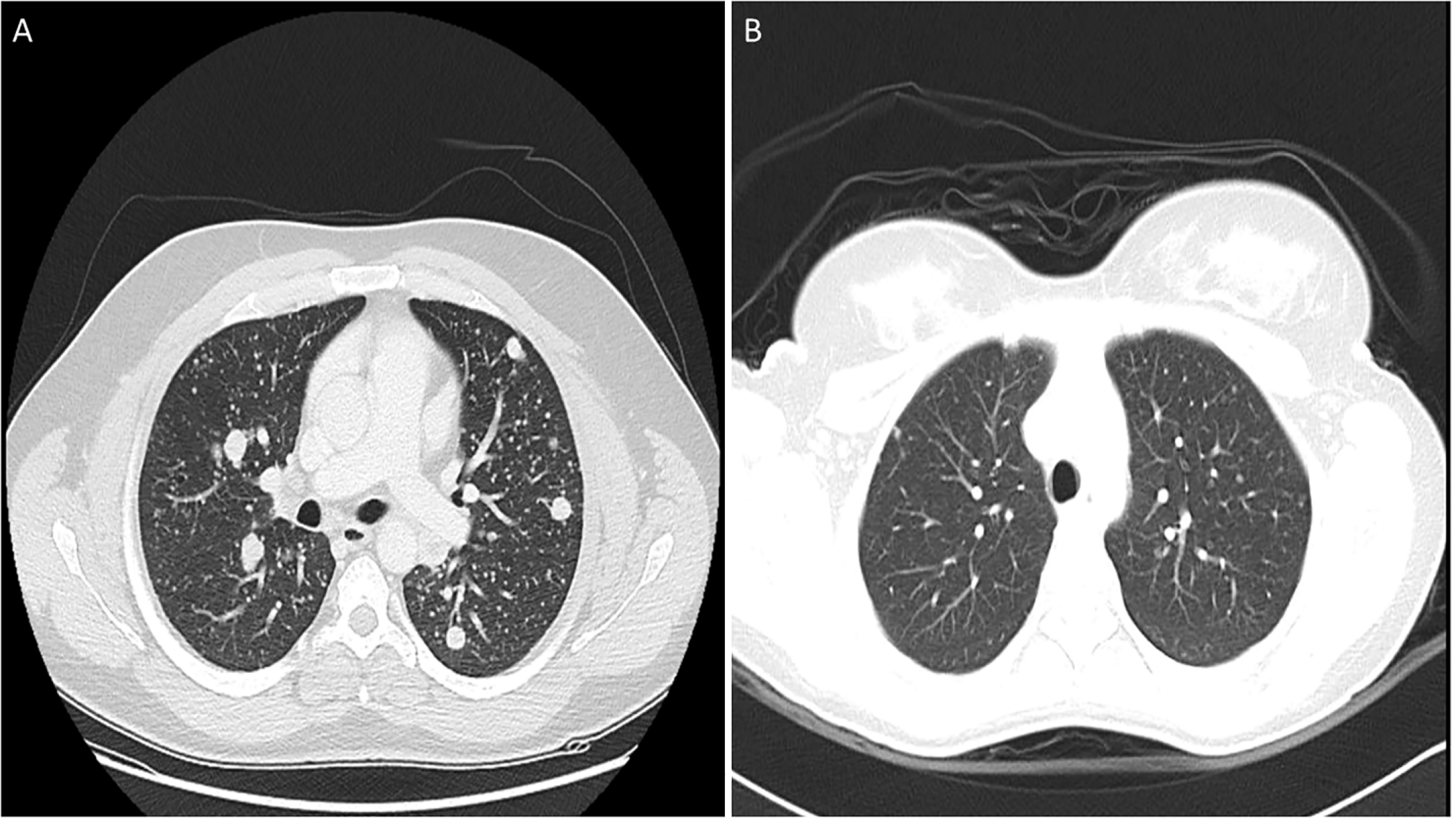
Computed tomography (CT) scan images of lung metastasis in pediatric differentiated thyroid carcinoma patients showing macronodular **(A)** and micronodular **(B)** pattern.

Mean age of the entire cohort was 14.9 years, with a male: female ratio of 1:3. Majority of the tumors were PTC (92.3%; 157/170). Regional lymph node metastasis (LNM) was noted in 63.5% (108/170) of cases and distant metastasis was present in 15.9% (27/170) (**Table 1**), with all of them being lung metastasis. Of the 27 cases with lung metastasis, 20 (74.1%) were identified within 6 months of diagnosis. Pattern of lung metastases were as follows: micronodular (≤ 1cm) in 22 patients, macronodular (> 1cm) in two patients and no apparent nodule (found only on DxWBS) in three patients (**Table 2**, **Figure 1**). Median follow-up duration was 8.1 years (range 0.8 – 32.9 years).

**Table 2:** Characteristics of lung metastasis: pattern, treatment and outcome.

Patient	Time to lung metastasis diagnosis (months)	Lung metastasis pattern	No. of RAI doses	Cumulative RAI doses	Structural outcome	Biochemical outcome
1	3	Macronodular	1	150.00	WBS+	Persistent
2	0	Micronodular	1	75.00	NED	Unknown
3	124	Micronodular	4	786.00	NED	Persistent
4	1	Micronodular	3	452.00	CT+	Persistent
5	27	Micronodular	1	200.00	CT+	NED
6	0	Micronodular	2	160.00	WBS+	Persistent
7	23	Micronodular	2	399.00	NED	Unknown
8	0	Micronodular	5	835.00	CT+	Persistent
9	14	Micronodular	2	300.00	NED	Persistent
10	32	Micronodular	3	466.79	CT+	Persistent
11	2	Micronodular	2	412.80	WBS+/CT+	Persistent
12	1	Macronodular	3	532.55	CT+	NED
13	0	Micronodular	5	563.00	WBS+	Persistent
14	0	Micronodular	4	557.00	CT+	Persistent
15	0	Micronodular	4	380.00	NED	Persistent
16	35	Micronodular	2	198.50	WBS+/CT+	Persistent
17	0	Micronodular	3	286.00	CT+	Persistent
18	1	Micronodular	4	619.00	CT+	Persistent
19	0	Micronodular	2	278.00	WBS+	Persistent
20	0	No apparent nodule	1	123.00	CT+	Persistent
21	4	Micronodular	1	125.00	NED	NED
22	61	No apparent nodule	3	400.00	WBS+/CT+	Persistent
23	1	No apparent nodule	1	40.00	CT+	NED
24	0	Micronodular	2	316.00	WBS+	Persistent
25	0	Micronodular	3	483.00	CT+	Persistent
26	0	Micronodular	3	258.00	WBS+	Persistent
27	6	Micronodular	4	650.00	CT+	Persistent
Overall median			3	380		

NED, No evidence of disease.

### Clinico-pathological characteristics associated with lung metastasis

**Figure 2:**
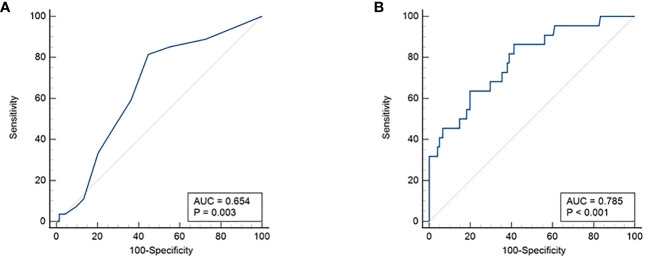
Receiver operating characteristics (ROC) curve. **(A)** Age cutoff of 15 years was found to be an accurate predictor of lung metastasis (area under the curve = 0.654, sensitivity = 70.4%, specificity = 54.6%, p = 0.0030). **(B)** Post-operative sTg >10ng/ml was shown to be an accurate predictor of lung metastasis (area under the curve = 0.785, sensitivity = 86.4%, specificity = 59.0%, p < 0.0001).

Lung metastasis was significantly associated with younger age (p = 0.0016): 77.8% (21/27) patients with lung metastasis being ≤ 15 years. ROC curve analysis also showed an age cutoff of 15 years was an accurate predictor of lung metastasis (area under the curve = 0.654, sensitivity = 70.4%, specificity = 54.6%, p = 0.0030) (**Figure 2A**). In addition, bilateral tumor (p = 0.0209), multifocality (p = 0.0352), extrathyroidal extension (p = 0.0015) and LNM (p = 0.0413) were also found to be significantly associated with lung metastasis. Elevated post-operative sTg was noted in 95.4% (21/22) of patients with lung metastasis (p = 0.0004) and it was shown to be an accurate predictor of lung metastasis by ROC curve analysis (area under the curve = 0.785, sensitivity = 86.4%, specificity = 59.0%, p < 0.0001) at a cutoff of >10ng/ml (**Figure 2B**). Older age (>15 years), absent extrathyroidal extension, absent LNM and low post-operative sTg also had negative predictive values > 90% (**Table 1**).

Although no mutations were detected in the *mitogen activated protein kinase* and *TERT* genes among the 16 lung metastatic cases tested, NTRK3 fusions were noted in 30.8% (4/13) and were significantly associated with lung metastasis (**Table 1**).

We further analyzed the clinico-pathological associations for *BRAF* mutation and *NTRK3* fusion in the entire cohort. *BRAF* mutation was significantly associated with older age (>15 years, p = 0.0058) and inversely associated with extrathyroidal extension (p = 0.0390), T stage (p = 0.0054) and lung metastasis (p = 0.0062) (**Supplementary Table 2**). *NTRK3* fusions were found to be significantly associated with only lung metastasis (p = 0.0379) (**Supplementary Table 3**).

### Detection of lung metastasis, treatment and response to therapy

74.1% (20/27) lung metastases were diagnosed within 6 months of thyroidectomy. Lung metastasis were diagnosed by preoperative CT in 17/27 (63%) patients, of whom 16 patients were also positive by pre-ablation ^123^I WBS. One patient had missing information about pre-ablation ^123^I WBS. Three patients had disease detected only by pre-ablation WBS. Six patients had a normal DxWBS but developed lung metastasis after a period ranging from 1-10 years, identified by surveillance ^123^I WBS, despite negative prior scans.

All patients with lung metastases were treated by RAI therapy. The median cumulative RAI activity was 380 mCi (range 75–835), and the median total number of RAI courses was 3 (range 1–5). At approximately one year after treatment, only one patient (3.7%) achieved an excellent response. Biochemical persistence was noted in 3 of 27 (11.1%) patients, structural persistence in 21 of 27 (77.7%) patients (including one death) and indeterminate in 2 of 27 (7.4%) patients (**Table 2**). Elevated post-operative sTg was the only risk factor found to be significantly associated with both biochemical persistence (with or without structural persistence (p = 0.0143)) and structural persistence (p = 0.0433). Neither RAI cumulative activity nor lung metastasis pattern had an impact on therapy response in this cohort (**Table 3**).

**Table 3:** Clinico-pathological characteristics associated with structural and biochemical persistence in patients with lung metastasis.

	Biochemical persistence			Structural persistence		
	Yes	No	p value	Yes	No	p value
≤ 15 years	17 (80.9%)	3 (75.0%)	1.0000	16 (76.2%)	5 (83.3%)	1.0000
16 – 18 years	4 (19.1%)	1 (25.0)		5 (213.8%)	1 (16.7%)	
Gender						
Male	7 (33.3%)	0	0.2945	6 (28.6%)	2 (33.3%)	1.0000
Female	14 (66.7%)	4 (100.0%)		15 (71.4%)	4 (66.7%)	
**Tumor diameter (cm), Mean ± SD**	4.23 ± 2.49	2.58 ± 0.30	0.2061	4.24 ± 2.40	2.85 ± 1.45	0.1926
Tumor laterality						
Unilateral	9 (42.3%)	2 (50.0%)	1.0000	10 (47.6%)	3 (50.0%)	1.0000
Bilateral	12 (57.1%)	2 (50.0%)		11 (52.4%)	3 (50.0%)	
Tumor focality						
Unifocal	6 (28.6%)	1 (25.0%)	1.0000	7 (33.3%)	1 (16.7%)	0.6334
Multifocal	15 (71.4%)	3 (75.0%)		14 (66.7%)	5 (83.3%)	
Extrathyroidal extension						
Present	15 (71.4%)	4 (100.0%)	0.5404	15 (71.4%)	6 (100.0%)	0.2843
Absent	6 (28.6%)	0		6 (28.6%)	0	
pT						
T1 and T2	10 (58.8%)	1 (33.3%)	0.5658	9 (52.9%)	3 (60.0%)	1.0000
T3 and T4	7 (41.2%)	2 (66.7%)		8 (47.1%)	2 (40.0%)	
pN						
N0	2 (10.0%)	0	1.0000	2 (10.0%)	0	1.0000
N1	18 (90.0%)	4 (100.0%)		18 (90.0)	6 (100.0%)	
Post-operative sTg						
High	18 (100.0%)	1 (33.3%)	0.0143	17 (100.0%)	3 (60.0%)	0.0433
Low	0	2 (66.7%)		0	2 (40.0%)	
Lung metastasis pattern						
Micronodular	18 (85.7%)	2 (50.0%)	0.3029	16 (76.2%)	6 (100.0%)	0.4162
Macronodular	1 (4.8%)	1 (25.0%)		2 (9.5%)	0	
No apparent nodule	2 (9.5%)	1 (25.0%)		3 (14.3%)	0	
**RAI cumulative activity (mCi), Mean ± SD**	413.05 ± 201.68	224.39 ± 215.59	0.1028	380.03 ± 204.51	344.17 ± 253.75	0.7220
*NTRK3* fusion						
Present	4 (40.0%)	0	0.5152	3 (33.3%)	1 (25.0%)	1.0000
Absent	6 (60.0)	2 (100.0%)		6 (66.7%)	3 (75.0%)	

### Risk factors for distant metastasis-free survival and risk stratification

**Figure 3:**
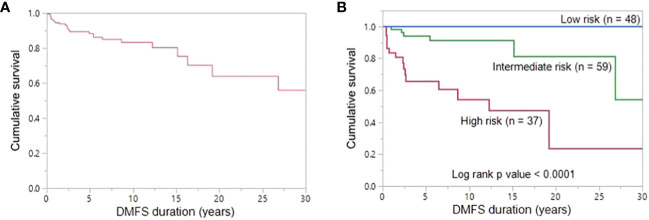
Distant metastasis-free survival. **(A)** The 5-, 10- and 20-year DMFS rates in the entire cohort (n = 170) were 89.3%, 83.2% and 63.8%, respectively. **(B)** The 20-year DMFS rates in the low-, intermediate-, and high-risk groups are 100.0%, 81.3% and 23.7%, respectively. DMFS is significantly better in low-risk group patients than in high-risk group patients and intermediate-risk group patients (P < 0.0001).

The 5-, 10- and 20-year DMFS rates were 89.3%, 83.2% and 63.8%, respectively (**Figure 3A**). On univariate analysis, younger age (≤ 15 years; p = 0.0230), extrathyroidal extension (p = 0.0386), LNM (p = 0.0252) and elevated post-operative sTg (p = 0.0055) were significantly related to DMFS (**Table 4**). However, on multivariate analysis, age younger than 15 years (Hazard ratio (HR) = 11.00; 95% confidence interval (CI) = 2.13 – 202.05; p = 0.0018) and high post-operative sTg (HR = 7.92; 95% CI = 1.57 – 144.23; p = 0.0079) were the only independent predictive markers of poor DMFS in this cohort (**Table 4**).

**Table 4:** Clinico-pathological factors associated with distant-metastasis-free survival.

Factors	Univariate			Multivariate		
	HR	95% CI	P value	HR	95% CI	P value
Histology, PTC	1.13	0.64 – 1.56	0.1133			
Male	0.74	0.21 – 2.09	0.5987			
Age ≤ 15 years	3.04	1.16 – 9.43	0.0230	11.00	2.13 – 202.05	0.0018
Bilateral tumors	1.82	0.72 – 4.52	0.1984			
Multifocal tumors	1.65	0.65 – 4.73	0.2971			
Extrathyroidal extension	2.77	1.05 – 8.61	0.0386	0.63	0.21 – 2.15	0.4449
Tumor size > 4cm	1.83	0.62 – 4.92	0.2587			
pN1	5.84	1.20 – 105.40	0.0252	3.02	0.58 – 55.64	0.2209
Post-operative Tg	8.29	1.67 – 150.07	0.0055	7.92	1.57 – 144.23	0.0079
Total thyroidectomy	5.62	0.55 – 17.90	0.4528			
Lymph node dissection	2.89	0.59 – 52.05	0.2253			

PTC, Papillary thyroid cancer; pN1, pathologic lymph node metastasis.

Based on the number of independent risk factors, patients were divided into 3 groups: low-risk (no risk factors); intermediate-risk (one risk factor); and high-risk (both risk factors). Risk stratification was performed for 144 patients for whom post-operative sTg data was available. 33.3% (48/144), 41.0% (59/144) and 25.7% (37/144) of patients were classified as low-, intermediate- and high-risk, respectively. 72.7% (16/22) of patients with lung metastasis were classified as high risk and 27.3% (6/22) were intermediate-risk. None of the lung metastasis patients were classified as low risk. 20-year DMFS rates in the low-, intermediate- and high-risk groups were 100.0%, 81.3% and 23.7% respectively (p < 0.0001) (**Figure 3B**).

We also analyzed the PFS with regards to predictive factors and risk stratification. Similar to DMFS, we found that age and high post-operative sTg were independent predictors of PFS (**Supplementary Table 1**) and risk stratification showed that 20-year PFS rates in the low-, intermediate- and high-risk groups were 84.1%, 73.7% and 19.2% respectively (p = 0.0005) (**Supplementary Figure 1**).

## Discussion

The management of CAYA DTC with DM is clinically challenging, given the favorable prognosis and protracted clinical course. In addition, there are concerns regarding the potential long-term complications of multiple treatments for CAYA DTC (15–17, 23, 24). In this study, we present a relatively large cohort of CAYA DTC from Middle Eastern ethnicity. DM was observed in 27 patients, and all were lung metastases. The prevalence of lung metastasis in our CAYA cohort is ~16% and is comparable with previous report conducted on series of pediatric DTC, where the prevalence of DM ranged from 15-25%, depending on the population studied and the cohort size (7, 12, 25–27).

We found that lung metastasis was significantly associated with age younger than 15 years, presence of LNM, bilateral tumors, multifocal tumors, extrathyroidal extension and post-operative sTg. There are few reports that investigated risk factors associated with DM in CAYA DTC, where they identified additional factors such as tumor size and T-stage as independent risk factors (12, 28, 29), whereas these were not significant factors in the present study. Interestingly, age older than 15 years, absence of LNM and low post-operative sTg levels had negative predictive value of more than 90% for lung metastasis. Post-operative sTg levels and age younger than 15 years were significant independent predictors for lung metastasis and both were shown to be an accurate predictor of lung metastasis by ROC curve analysis. Indeed, in concordance with our findings, a recent study showed that sTg was an independent predictor of 1-year treatment response and final outcome in pediatric DTC (30).

Most lung metastasis 20/27 (74%) were identified within 6 months from thyroidectomy. Pattern of lung metastasis were mostly micronodular (22/27; 81.5%), with macronodular pattern seen in 2 cases and no apparent nodule (negative by CT Scan and detected by pre-ablation WBS) in 3 patients.

Genetic analysis for *mitogen activated protein kinase* and *TERT* mutations were assessed in 117/170 and 106/170 patients, respectively. None of the DM cases were positive for these mutations. *NTRK3* fusion was assessed successfully in 68/170 cases. 30.8% (4/13) of DM patients tested were found positive for *NTRK3* fusion, compared to 7.3% (4/55) of non-metastatic cases. *RET* fusions were not analyzed due to technical reasons. Despite the small number of cases tested for *NTRK3* fusion, the significant association between *NTRK3* fusion and lung metastasis further highlights our previous finding (22) that *NTRK3* plays an important role in aggressive pediatric DTC and confirms the notion that pediatric tumors are commonly driven by oncogenic fusions involving *NTRK3* and less commonly driven by *BRAF* and *TERT* mutations. Indeed, this is in concordance with recent studies that have shown NTRK fusions to be enriched in pediatric lung metastasis cases, suggesting that NTRK fusions may be associated with tumor progression in pediatric DTC (31, 32).

Lung metastases were diagnosed by preoperative CT in 17/27 (63%) patients, of whom 16 patients were also positive by pre-ablation ^123^I WBS. One patient had missing information about pre-ablation ^123^I WBS. Three patients had disease detected only by pre-ablation WBS. All of these three patient were classified as high risk according to ATA risk stratification. Six patients had normal diagnostic WBS but developed lung metastasis after 1-10 years, identified by surveillance ^123^I WBS, despite negative prior scans. These finding highlight the importance of multimodality imaging, especially in patients at high risk for recurrence.

All patients with DM were treated by RAI therapy. The overall response was evaluated using the ATA guidelines (19). After a median follow-up of 12.1 years, only one patient (3.7%) achieved an excellent response. Similarly low excellent response rates were reported previously in patients under 20 years (9, 12, 33). Despite a high rate of structural persistent disease, overall survival rate was excellent (96.3%).

We did not identify any association between cumulative RAI activity (median = 380 mCi), number of RAI courses (median = 3) or pattern of lung metastasis and structural outcome. This could suggest that higher RAI doses might not improve the outcome of these patients and different systemic therapy might have to be considered for those with progressive DM.

In this study, we also estimated the DMFS for all patients. The 5-, 10- and 20-year DMFS rates were 89.3%, 83.2% and 63.8%, respectively. Multivariate analysis showed that age younger than 15 years and high post-operative sTg are the only predictive markers of poor DMFS in this cohort. Furthermore, based on the number of risk factors related to poor DMFS, we divided the patients into low-risk, intermediate-risk and high-risk groups. Interestingly, DMFS was significantly different among the three risk groups. The 20-year DMFS group rate in low-, intermediate-, and high-risk groups were 100.0%, 81.3% and 23.7%, respectively. Interestingly, all three patients with DM detected only by ^131^I WBS were in the high-risk group according to our proposed risk stratification. The low number of patients might limit the applicability of our risk stratification but should encourage a risk-adapted management for CAYA with DTC.

This study had certain limitations. Firstly, the retrospective nature of this study from a single institution is prone to bias. Secondly, the molecular alterations were not analyzed on the whole cohort samples and hence the results should be interpreted with caution. Thirdly, risk stratification was done in a limited number of patients from specific ethnicity, which precludes generalizability. Larger studies from other populations should be encouraged before applying the stratification.

In conclusion, in this cohort of CAYA DTC, lung metastases were associated with younger age, extrathyroidal extension, bilateral tumors, LN disease, a high post-operative sTg and *NTRK3* fusions. Excellent response is rare and multiple RAI courses do not affect the outcome in these patients. Younger age and post-operative sTg have a potential role in stratifying CAYA DTC with worse DMFS.

## Data availability statement

The original contributions presented in the study are included in the article/**Supplementary Material**. Further inquiries can be directed to the corresponding author.

## Ethics statement

The studies involving humans were approved by Research Advisory Council (RAC), King Faisal Specialist Hospital and Research Centre (Riyadh, Saudi Arabia). The studies were conducted in accordance with the local legislation and institutional requirements. The ethics committee/institutional review board waived the requirement of written informed consent for participation from the participants or the participants’ legal guardians/next of kin because since only retrospective patient data were used, the Research Advisory Council (RAC) provided waiver of consent under project RAC # 221 1168 and # 2110 031.

## Author contributions

Study concept and design: KA-K, SP, AS. Executed the study: SP, AS, PA, NS, MA-R, WA-H, SA-S, FA-D. Statistical analysis: ZQ, KS. Drafting the article: KA-K, AS, SP. Critical revision of the article for important intellectual content, writing of the article, and approval of the final version: KA-K, SP, AS, PA, NS, MA-R, WA-H, ZQ, KS, SA-S, FA-D. All authors contributed to the article and approved the submitted version.
