# Supplementary material for: Predictive risk factors for distant metastasis in pediatric differentiated thyroid cancer from Saudi Arabia
Source: Front Endocrinol (Lausanne). 2023 Oct 6;14:1228049. doi: 10.3389/fendo.2023.1228049 (PMC10587684; doi:10.3389/fendo.2023.1228049)
Supplement: Supplementary file 1 [file DataSheet_1.docx]

**Supplementary Table 1. Clinico-pathological factors associated with progression-free survival**

|  | **Univariate** | | | **Multivariate** | | |
| --- | --- | --- | --- | --- | --- | --- |
| **Factors** | **HR** | **95% CI** | **P value** | **HR** | **95% CI** | **P value** |
| Histology, PTC | 1.77 | 0.38 – 31.44 | 0.5388 |  |  |  |
| Male | 0.49 | 0.18 – 1.09 | 0.0837 |  |  |  |
| Age ≤ 15 years | 2.20 | 1.13 – 4.52 | 0.0194 | 3.09 | 1.30 – 8.61 | 0.0097 |
| Bilateral tumors | 2.22 | 1.16 – 4.29 | 0.0167 | 1.29 | 0.59 – 2.95 | 0.5252 |
| Multifocal tumors | 1.59 | 0.82 – 3.29 | 0.1753 |  |  |  |
| Extrathyroidal extension | 1.93 | 0.98 – 4.00 | 0.0560 |  |  |  |
| Tumor size > 4cm | 2.29 | 1.14 – 4.45 | 0.0206 | 2.16 | 0.99 – 4.62 | 0.0531 |
| pN1 | 5.07 | 1.54 – 31.29 | 0.0044 | 1.60 | 0.42 – 10.42 | 0.5242 |
| Post-operative Tg | 4.60 | 1.63 – 19.20 | 0.0021 | 4.33 | 1.24 – 27.37 | 0.0187 |
| Total thyroidectomy | 2.32 | 0.83 – 9.62 | 0.1161 |  |  |  |
| Lymph node dissection | 1.62 | 0.58 – 6.73 | 0.3912 |  |  |  |

PTC – Papillary thyroid cancer; pN1 – pathologic lymph node metastasis; Tg - thyroglobulin

**Supplementary Table 2. Clinico-pathological characteristics and associations of CAYA DTC with and without *BRAF* mutation**

|  | **Total n = 117** | ***BRAF* mutant n = 28** | ***BRAF* wildtype n = 89** | **p value** |
| --- | --- | --- | --- | --- |
| **Age at diagnosis, years (mean ± SD)** | 14.8 ± 3.1 | 16.3 ± 2.4 | 14.3 ± 3.2 | 0.0029 |
| ≤ 15 years | 60 (51.3%) | 8 (28.6%) | 52 (58.4%) | 0.0058 |
| 16 – 18 years | 57 (48.7%) | 20 (71.4%) | 37 (41.6%) |  |
| **Gender** |  |  |  |  |
| Male | 32 (27.4%) | 6 (21.4%) | 26 (29.2%) | 0.4202 |
| Female | 85 (72.6%) | 22 (78.6%) | 63 (70.8%) |  |
| **Tumor diameter (cm)** | 3.3 ± 1.8 | 2.9 ± 1.2 | 3.4 ± 1.9 | 0.1926 |
| **Histologic subtype** |  |  |  |  |
| FTC | 8 (6.8%) | 0 | 8 (9.0%) | 0.1956 |
| PTC | 109 (93.2%) | 28 (100.0%) | 81 (91.0%) |  |
| **Tumor laterality** |  |  |  |  |
| Unilateral | 74 (63.2%) | 19 (67.9%) | 55 (61.8%) | 0.5619 |
| Bilateral | 43 (36.8%) | 9 (32.1%) | 34 (38.2%) |  |
| **Tumor focality** |  |  |  |  |
| Unifocal | 55 (47.0%) | 12 (42.9%) | 43 (48.3%) | 0.6138 |
| Multifocal | 62 (53.0%) | 16 (57.1%) | 46 (51.7%) |  |
| **Extrathyroidal extension** |  |  |  |  |
| Present | 60 (52.6%) | 10 (35.7%) | 50 (58.1%) | 0.0390 |
| Absent | 54 (47.4%) | 18 (64.3%) | 36 (41.9%) |  |
| **pT** |  |  |  |  |
| T1 and T2 | 78 (68.4%) | 25 (89.3%) | 53 (61.6%) | 0.0054 |
| T3 and T4 | 36 (31.6%) | 3 (10.7%) | 33 (38.4%) |  |
| **pN** |  |  |  |  |
| N0 | 26 (24.8%) | 7 (26.9%) | 19 (24.1%) | 0.7685 |
| N1 | 79 (75.2%) | 19 (73.1%) | 60 (75.9%) |  |
| **Lung metastasis** |  |  |  |  |
| Present | 19 (16.2%) | 0 | 19 (21.4%) | 0.0062 |
| Absent | 98 (83.8%) | 28 (100.0%) | 70 (78.6%) |  |

**Supplementary Table 3. Clinico-pathological characteristics and associations of CAYA DTC with and without *NTRK3* fusion**

|  | **Total n = 68** | ***NTRK3* fusion present n = 8** | ***NTRK3* fusion absent n = 60** | **p value** |
| --- | --- | --- | --- | --- |
| **Age at diagnosis, years (mean ± SD)** | 14.6 ± 3.1 | 13.4 ± 2.4 | 14.8 ± 3.2 | 0.2382 |
| ≤ 15 years | 37 (54.4%) | 6 (75.0%) | 31 (51.7%) | 0.2752 |
| 16 – 18 years | 31 (45.6%) | 2 (25.0%) | 29 (48.3%) |  |
| **Gender** |  |  |  |  |
| Male | 18 (26.5%) | 3 (37.5%) | 15 (25.0%) | 0.4279 |
| Female | 50 (73.5%) | 5 (62.5%) | 45 (75.0%) |  |
| **Tumor diameter (cm)** | 3.4 ± 1.7 | 3.9 ± 2.3 | 3.3 ± 1.6 | 0.3484 |
| **Histologic subtype** |  |  |  |  |
| FTC | 1 (1.5%) | 0 | 1 (1.7%) | 1.0000 |
| PTC | 67 (98.5%) | 8 (100.0%) | 59 (98.3%) |  |
| **Tumor laterality** |  |  |  |  |
| Unilateral | 40 (58.8%) | 3 (37.5%) | 37 (61.7%) | 0.2590 |
| Bilateral | 28 (41.2%) | 5 (62.5%) | 23 (38.3%) |  |
| **Tumor focality** |  |  |  |  |
| Unifocal | 28 (41.2%) | 3 (37.5%) | 25 (41.7%) | 1.0000 |
| Multifocal | 40 (58.8%) | 5 (62.5%) | 35 (58.3%) |  |
| **Extrathyroidal extension** |  |  |  |  |
| Present | 38 (55.9%) | 6 (75.0%) | 32 (53.3%) | 0.2880 |
| Absent | 30 (44.1%) | 2 (25.0%) | 28 (46.7%) |  |
| **pT** |  |  |  |  |
| T1 and T2 | 47 (71.2%) | 4 (57.1%) | 43 (72.9%) | 0.4012 |
| T3 and T4 | 19 (28.8%) | 3 (42.9%) | 16 (27.1%) |  |
| **pN** |  |  |  |  |
| N0 | 13 (19.1%) | 0 | 13 (21.7%) | 0.3372 |
| N1 | 55 (80.9%) | 8 (100.0%) | 47 (78.3%) |  |
| **Lung metastasis** |  |  |  |  |
| Present | 13 (19.1%) | 4 (50.0%) | 9 (15.0%) | 0.0379 |
| Absent | 55 (80.9%) | 4 (50.0%) | 51 (85.0%) |  |


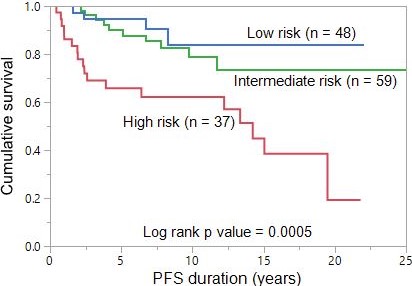


**Supplementary figure 1:** **Progression-free survival (PFS).** The 20-year PFS rates in the low-, intermediate-, and high-risk groups are 84.1%, 73.7% and 19.2%, respectively. PFS is significantly better in low-risk group patients than in high-risk group patients and intermediate-risk group patients (P = 0.0005).
